# Laying Open and Curettage under Local Anesthesia to Treat Pilonidal Sinus: Long-Term Follow-Up in 111 Consecutively Operated Patients

**DOI:** 10.3390/clinpract11020028

**Published:** 2021-04-01

**Authors:** Pankaj Garg, Vipul D. Yagnik

**Affiliations:** 1 Chief Colorectal Surgeon, Indus International Hospital, Mohali 140507, India; 2 Nishtha Surgical Hospital and Research Centre, Patan 384265, Gujarat, India; vipul.yagnik@gmail.com or

**Keywords:** pilonidal, lay open, deroofing, curettage, sinus

## Abstract

(1) Background: Several techniques for the treatment of pilonidal sinus disease (PSD) are in vogue, though none have emerged as the gold standard. Laying open (deroofing) and curettage under local anesthesia is one of the most straightforward procedures to treat PSD. In this study, the long-term follow-up in a large series was analyzed. (2) Methods: The laying open approach was performed for all types of consecutive PSD patients—simple, complicated, and abscess. The primary outcome parameter of the study was the healing rate. The secondary outcome parameters were operating time, hospital stay, time to resumption of normal work, and healing time. (3) Results: 111 (M/F–92/19, mean age-22.9 ± 5.7 years) consecutive patients were operated on and followed for 38 months (6–111 months). Of these, 24 had pilonidal abscesses, 87 had chronic pilonidal disease, while 22 had recurrent disease. Operating time and hospital stay were 24 ± 7 min and 66 ± 23 min, respectively. On average, patients could resume normal work in 3.6 ± 2.9 days and the healing time was 43.8 ± 7.4 days. Three patients were lost to follow-up. Complete resolution of the disease occurred in 104/108 (96.3%) patients, while 4 (3.7%) had a recurrence. One recurrence was due to a missed tract, while three recurrences presented after complete healing had occurred. Two patients with recurrence were operated on again with the same procedure, and both healed completely. Thus, the overall success rate of this procedure was 98.1% (106/108) with a recurrence rate after first surgery of 3.7% over a median follow-up of 38 months. (4) Conclusions: Pilonidal disease managed by laying open (deroofing) with curettage under local anesthesia is associated with a high cure rate. This procedure is effective in treating all kinds of pilonidal disease (simple, complicated, and abscess).

## 1. Introduction

Though pilonidal sinus disease (PSD) was first defined as far back as 1880 [1], its treatment is still not definitive. Several treatment options have been advocated for PSD, including incision and drainage for acute abscess [2], wide excision and leaving it open to heal by secondary intention [3], or wide excision followed by closure of the resulting wound. The wound closure may be in the midline [4], or a flap may be utilized with the twin purposes of releasing tension in the wound and flattening the contour of the buttocks. Several types of flaps have been used for this purpose, including Karydakis flap [5], Z-plasty [6], Limberg flap [7], Bascom flap [8], etc. Of late, laser and endoscopic procedures have been described to treat PSD with a high success rate [9,10]. Although these extensive procedures increase the magnitude of the surgery and the risk of tissue loss, none of them are established as the gold standard procedure to treat PSD [7,8].

The laying open (deroofing) with curettage under local anesthesia (LOCULA) procedure is a simple procedure with several distinct advantages [11]. Our initial short-term experience with this procedure in 33 patients was reported in 2015 [11]. In the present study, the long-term success rate of this procedure in a much larger series (111 patients) is evaluated.

## 2. Materials and Methods

### 2.1. Study Population

In a prospective cohort, all consecutive patients suffering from sacrococcygeal pilonidal disease operated by LOCULA procedure between 2011 to 2020 at a single center were included in the study. A single surgeon performed all the surgeries. Patients with all types of PSD—chronic disease, recurrent disease, and PSD presenting with an abscess—were included. The primary outcome parameter of the study was complete healing of the pilonidal sinus. The secondary outcome parameters were operating time, hospital stay, and resumption of everyday activities. Informed written consent was taken from every patient. Approval was granted by the Indus International Hospital-Institute Ethics Committee (IIH-IEC) via approval number: Indus hospital/EC/01-11.

### 2.2. Surgical Procedure

**Figure 1:**
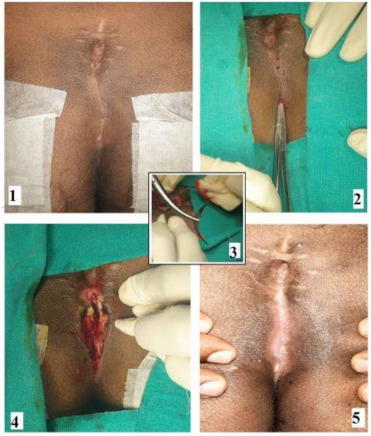
LOCULA (lay open with curettage under local anesthesia) in a 27-year-old male patient with recurrent pilonidal sinus who had undergone wide excision and midline closure previously. (**1**). Preoperative photo, (**2**) A curved artery forceps is inserted into the sinus cavity, (**3**) A bunch of hairs being removed from the cavity, (**4**) Final postoperative wound, (**5**) Complete healing of the wound after seven weeks.

The procedure was performed under local anesthesia in a day-care setting. The patient was placed in the prone position, and an adhesive tape was used to spread the buttocks. Twenty ml of 2% lignocaine with adrenaline (1:100,000) was used for local anesthesia. The anesthetic solution was injected all around the sinus tract intended to be deroofed. Even in patients with acute abscess, anesthetic solution infiltration around the abscess led to adequate anesthesia with satisfactory pain relief. Initially, it was expected that local anesthesia might not work in patients with acute pilonidal abscess. However, it proved effective in every patient, and general or spinal anesthesia was not required. The sinus opening was probed gently to gauge the direction and length of the tract. A curved artery-forceps was inserted in the sinus tract, and the overlying tissue was incised with electrocautery (Figure 1).

All the branches were identified and laid open in continuity. The debris and hair were extracted from the sinus, and infected granulation tissue was removed thoroughly by rubbing with dry gauze or with the help of a curette (Figure 1). The overlying skin edges were trimmed to convert the sinus cavity into a saucer-shaped wound. The wound was again checked thoroughly for any branches. The lateral wall and the base of the sinus were left intact, but their epithelium was cauterized. No marsupialization or closure of the wound was done. The wound was packed lightly with gauze.

After the procedure, the patient walked to the recovery room and was instructed to lie in a supine position putting full bodyweight on the operated site for half an hour. After this, if the wound-site dressing did not show any active bleeding, the patient was discharged from the hospital with instructions to resume his/her daily routine. However, avoidance of strenuous activities for a week was recommended.

### 2.3. Follow-Up

The dressing was removed on the next (first postoperative) day in the out-patient department. The wound was rubbed gently with dry gauze. This was done once or twice a day so that the wound edges did not adhere, and healing occurred by secondary intention. Follow-up was done weekly till the wound healed completely.

Once wound healing was complete, the patient was instructed to regularly remove hairs all around the wound and to apply talcum powder around the scar in the intergluteal cleft for a minimum of five years [11]. (India being a hot and humid country, most patients report increased sweating and moistness in the intergluteal region. The persistent moisture macerates the skin and may predispose to the development of pilonidal sinus. Talcum powder application helps keep the intergluteal area dry). The patient was also instructed to report in case there was any pain, swelling, or pus discharge from the scar.

## 3. Results

A total of 111 (M/F–92/19, mean age: 22.9 ± 5.7 years, range: 15–39 years) consecutive patients were operated on over nine years and followed up for a median of 38 months (6–111 months). Of these, 24 (21.6%) patients had a pilonidal abscess, 87 (78.4%) were suffering from chronic pilonidal disease, 22 (19.8%) had recurrent disease (12, after wide excision with open healing; 3, after wide excision and midline closure; and 7, after wide excision and flap surgery). (Table 1).

Operating time and hospital stay were 24 ± 7 min and 66 ± 23 min, respectively. On average, patients could resume regular work in 3.6 ± 2.9 days and the healing time was 43.8 ± 7.4 days. Three patients were lost to follow-up. Complete healing of the disease was seen in 104/108 (96.3%) patients, while 4 (3.7%) had a recurrence (Table 2).

One recurrence was due to a missed tract, while three recurrences were seen well after complete healing of the wound. Two of the four patients with recurrence were operated on again with the same procedure (LOCULA), and both healed. Thus, the overall success rate of this procedure was 106/108 (98.1%) with a recurrence rate of 3.7% after first surgery over a median follow-up of 38 months. (Table 2). In abscess patients (n = 24), LOCULA was performed as the single-stage definitive procedure, and all the patients healed. In 5/108 (4.6%) patients, minor bleeding occurred from the postoperative wound and it was controlled easily by gentle pressure with a gauze pad. No patient required any surgical intervention for the bleeding.

## 4. Discussion

The present study is one of the largest studies till date, which demonstrates that laying open and curettage of the pilonidal cavity under local anesthesia (LOCULA) is associated with a remarkably high success rate on long-term follow-up. The study is not novel as the LOCULA procedure and its distinct advantages over other techniques have been described previously [11]. Still, this procedure is not widely used. In addition to being one of the largest studies with the LOCULA procedure, this study highlights its efficacy on a long-term basis. Therefore, the present study would help establish this technique as the frontline procedure for management of all pilonidal sinus disease types.

In the LOCULA procedure, no attempt is made to excise the sinus. Only deroofing (laying open) is performed, and the overhanging margins are partially trimmed to create a saucer-shaped wound (Figure 1). This helps prevent adherence of wound edges, thus promoting healing by secondary intention. As no excision is done, the procedure is very simple, less time-consuming, associated with little bleeding, and the resulting wound is relatively small (Figure 1). Thus, postoperative pain is minimal, and most patients do not require any analgesics three days after the procedure.

As demonstrated by this study, there are distinct advantages of the LOCULA procedure. It can be done under local anesthesia, is a simple procedure requiring short operating time, needs only a small incision as the pilonidal sinus/cavity is not excised but merely deroofed (Figure 1), does not require admission, allows early resumption of everyday activities, is associated with a high cure rate (98% in the present study), and can be done in all types of PSD [simple as well as complicated (recurrent, deep, multiple branches etc.)], can be done as a first-line definitive procedure in cases of pilonidal sinus with abscess, can be repeated easily in case of a recurrence, is easy to learn and replicate, does not require any expensive tools like laser or endoscopic equipment, and does not flatten the contour of the upper buttocks.

However, there were two distinct disadvantages of the LOCULA procedure. First, wound healing takes approximately 5–8 weeks. However, in reality, this prolonged healing duration does not bother the patient much, as it does not interfere with their normal routine or professional work. Second, local hygiene and care (hair removal and powder application) are required for a few years.

The recurrence rate of 1.9% after LOCULA is comparable to the recurrence rate reported after other procedures routinely performed to treat PSD: 0–11.9% for excision with open healing [12,13], 0–7.1% for excision with marsupialization [12,13], 0–20% for excision with midline closure [12,13,14], 0–11% for excision with off midline closure with different flaps [12,13,14], 2–9% with laser treatment [9] and 1–8% with endoscopic procedures [10].

LOCULA should not be confused with simple incision and drainage of an acute pilonidal abscess. The latter was associated with a recurrence rate of up to 24% [2,15,16,17]. However, when the cavity was curetted along with incision and drainage, the recurrence rate reduced significantly. A large study (150 patients) with long-term follow-up (65 months) demonstrated that the cure rate rose from 46% to 90% when curettage was added to simple incision and drainage of pilonidal abscess [17]. The reason was that thorough curettage of the sinus cavity removed all the debris and infected granulation tissue and helped identify any side branches/extensions, which were then laid open.

Wide excision had been advocated for PSD for several years. An important point to debate is why a simple infected wound should undergo excision and that too wide excision? PSD is not a malignancy, and wide excision is perhaps not warranted [11]. If wound closure is contemplated after wide excision of PSD, a variable degree of tension in the closed wound is inevitable because of tissue deficit. Consequently, a flap is usually required to allow wound closure without tension. PSD is like any ordinary subcutaneous abscess with a ‘slightly different etiology’. Wide excision and primary closure are not required in any subcutaneous abscess anywhere else in the body; similarly, wide excision is perhaps not needed in PSD.

Various causative factors such as hairs, prolonged sitting, overweight, etc., predispose to PSD development. We suspect that excessive sweating may be one of the causative factors as well, but there is currently no proof. Hair is one of the leading causative factors for PSD [18,19,20,21]. Conventionally, it is believed that hair fragments from the intergluteal fold are responsible for PSD [3,8]. However, the latest research on the origin of the causative hair suggests that occipital hair could be responsible for PSD rather than hair from the intergluteal fold or lumbar region [22,23,24]. These two causative factors (hair and suspectedly sweating) are important because they are the only two modifiable risk factors and can help prevent the disease in the future. These can be easily modified by regular removal of hair (or decreasing hair growth by the laser procedure) and frequent talcum powder application in the intergluteal cleft. These lifestyle changes need to be maintained for at least five years after the procedure. This protocol was followed in this study, and it was significantly effective in preventing recurrence in the healed patients on long-term follow-up. A flap procedure can circumvent the need for these precautions as it flattens the upper buttocks’ contour. Once the natal cleft is flattened, accumulation of hair and sweat in the intergluteal cleft is drastically reduced, and hence the likelihood of recurrence is minimized. But, a significant proportion of patients prefer to prevent disease recurrence by countering predisposing factors rather than by altering their anatomy [18]. This was highlighted in a recent study in which 97.1% of patients did not prefer a flap procedure but opted for techniques that preserved the contour of the upper buttocks [18]. They did not mind hair clearing and powder application to prevent the disease recurrence [18]. The main reasons for not preferring procedures that flatten the upper buttocks (flap procedures) were the permanent change in anatomy, more prominent scar, and loss of cosmesis (since the presence of a cleft in upper buttocks may have cosmetic value). Since PSD is primarily a disease of young people [18], it is understandable that many young patients would not agree to flatten their gluteal contour.

With the advent of endoscopic surgery in other parts of the body, endoscopy has also been performed in PSD [10]. The use of endoscopic equipment can be justified when the surgery is performed in a deep plane, but when the pathology is just skin-deep, the use of endoscopic procedures seems illogical. It is in effect advocating an endoscopic approach to drain and cauterize a small subcutaneous abscess. Moreover, endoscopy increases the cost without substantially increasing the success rate [19].

As newer technologies using lasers were developed, these were also utilized in the treatment of PSD [10]. This unnecessarily increased the cost of the treatment. The use of expensive equipment (as required in laser procedure) is perhaps not needed, as the same purpose (ablation of the infected epithelium of the pilonidal tract/cavity) can be conveniently achieved with electrocautery at nominal cost [10,19].

The study had limitations. First, the study would have been much more informative if there were two comparative groups with properly calculated sample size in each group, and the success rate, healing time, and complications between them could be statistically compared. Second, though the procedure was associated with minimal pain and most patients could resume work within 3–4 days, the pain assessment should have been done by an objective pain scoring system and a patient satisfaction survey. That would have enhanced the objectivity of the results of the study.

## 5. Conclusions

This is one of the largest studies performed to treat PSD with an extended follow-up, demonstrating that laying open and curettage under local anesthesia has a high overall cure rate of 98.1% with a recurrence rate of 3.7% after first surgery over a median follow-up of 38 months. It is effective in simple and complicated PSD (recurrent, multiple branches, or associated abscess). This procedure can be done under local anesthesia as day-care surgery and is associated with minimal morbidity. Apart from this, it can be repeated easily in case of a recurrence, is simple, easy to learn and replicate, does not require any expensive equipment, and does not alter the buttock contour. Randomized controlled trials are needed to corroborate the findings of the study.

The present study demonstrates that laying open and curettage of pilonidal sinus disease under local anesthesia (LOCULA) is associated with a remarkably high success rate on long-term follow-up.

## Figures and Tables

**Table 1:** Demographic data & patient characteristics.

Parameter	N = 111
Age	22.9 ± 5.7 years
Sex	92/19
Follow-up Median (months)	36 (4–111)
Anesthesia	LA
Inclusion criteria	Chronic, Recurrent, Abscess
Exclusion criteria	Refused consent
Recurrent	22 (19.8%) (12-After wide excision with open healing, 7-After flap surgery, 3-After wide excision and midline closure)
Abscess	24 (21.6%)

**Table 2:** Results.

Parameter	N = 111
Operating time	24 ± 7 min
Hospital stay	66 ± 23 min
Resume normal work	3.6 ± 2.9 days
Healing time	43.8 ± 7.4 days
Recurrence	4 (3.7%)
Healing rate:	
After first surgery	104/108 (96.3%)
After second surgery (n = 2)	106/108 (98.1%)
Healing in Abscess patients (n = 24)	24/24 (100%)
Complications-Minor bleeding	5/108 (4.6%)

## Data Availability

The data presented in this study are available on request from the corresponding author. The data are not publicly available due to privacy and ethical issues.
